# Identifying Future Study Designs for Mental Health and Social Wellbeing Associated with Diets of a Cohort Living in Eco-Regions: Findings from the INSUM Expert Workshop

**DOI:** 10.3390/ijerph20010669

**Published:** 2022-12-30

**Authors:** Friederike Elsner, Lea Ellen Matthiessen, Dominika Średnicka-Tober, Wolfgang Marx, Adrienne O’Neil, Ailsa A. Welch, Richard Peter Hayhoe, Suzanne Higgs, Marja van Vliet, Ephimia Morphew-Lu, Renata Kazimierczak, Rita Góralska-Walczak, Klaudia Kopczyńska, Thea Steenbuch Krabbe Bruun, Beatriz Philippi Rosane, Susanne Gjedsted Bügel, Carola Strassner

**Affiliations:** 1 Department of Food, Nutrition, Facilities, FH Münster University of Applied Sciences, 48149 Muenster, Germany; 2 Department of Nutrition, Exercise and Sports, University of Copenhagen, 1958 Frederiksberg, Denmark; 3 Department of Functional and Organic Food, Institute of Human Nutrition Sciences, Warsaw University of Life Sciences, 02-776 Warsaw, Poland; 4 Food & Mood Centre, IMPACT—The Institute for Mental and Physical Health and Clinical Translation, School of Medicine, Barwon Health, Deakin University, Geelong, VIC 3220, Australia; 5 Norwich Medical School, University of East Anglia, Norfolk NR4 7TJ, UK; 6 School of Allied Health, Faculty of Health, Education, Medicine and Social Care, Anglia Ruskin University, Chelmsford CM1 1SQ, UK; 7 School of Psychology, University of Birmingham, Birmingham B15 2TT, UK; 8 Institute for Positive Health, 3521 AL Utrecht, The Netherlands; 9 The Center for Nutritional Psychology, San Jose, CA 95129, USA

**Keywords:** mental and social health, wellbeing, sustainable and healthy diet, Eco-Regions, sustainable food systems

## Abstract

Diets influence our mental health and social wellbeing (MHSW) in multiple ways. A rising community concept, Eco-Regions, has gained interest. The research project “Indicators for assessment of health effects of consumption of sustainable, organic school meals in Ecoregions” (INSUM) aims to develop future-oriented research approaches to measure the potential health effects of more sustainable and healthy diets. This first part of the project focuses on MHSW with the goal to identify suitable study designs and indicators. The methodology is based on a 2-day workshop with an interdisciplinary group of experts. This paper describes commonly applied research methods on the nexus between diet and MHSW as presented by the experts and summarises key points from the discussions. The results show that the dominating tool to investigate MSHW is questionnaires. Questionnaires vary largely depending on the research design, such as participants or distribution channels. Cohort studies addressing families and including in-depth interventional and/or experimental studies may be suitable for an Eco-Region investigation. Those MHSW studies can be conducted and combined with measurements of somatic health effects. We conclude that indicators should be seen as complementary rather than independent. Explorative research designs are required to investigate complex Eco-Regions.

## 1. Introduction and Aim of the Workshop

Current environmental pressure on and from the food system demands more sustainable ways of living. Food systems are connected to a multiplicity of health challenges, including social inequalities regarding access to healthy food [1]. Thus, food systems affect human health, mentally, socially, and physically [2]. Eco-Regions (or Bio-Districts or Organic Districts) are community concepts addressing issues related to sustainable development, including the full life cycle of food products (i.e., local value chains) and utilising organic food and farming practices and principles [3,4]. For instance, the Cilento Eco-Region in Italy is built around three dimensions, the social, the environmental and the economic. Eco-Regions suggest an innovative solution for sustainable, integrated and participatory territorial development [4]. The International Network of Eco Regions (INNER) describes the concept of Eco-Regions as a territorial approach to rural development utilising organic food and farming practices and principles [5]. Eco-Regions exist around the world, approximately 60 are placed in Europe (REF MAP). The potential health effects of living in these regions have yet to be investigated.

The research project “Indicators for assessment of health effects of consumption of sustainable, organic school meals in Ecoregions” (INSUM) aims to define the most suitable indicators and biomarkers to be used for future research on the diet and health nexus in Eco-Regions (http://www.insum.eu (accessed on 21 December 2022)). School meals are captive catering situations where all or most pupils participate in the food service provision. INSUM’s scope is not exclusively limited to the population group pupils and, thus, can be extended to other population groups. The main emphasis lies on indicators to measure the health effects of dietary changes rather than on traditional disease-risk markers. The assessment of health and resilience goes beyond traditional measurements of disease-risk markers that underlie metabolic imbalances [6]. INSUM is divided into different parts: the first part aims to assess the usefulness of current mental health and social wellbeing (MHSW) indicators in relation to diet. The second part addresses somatic health indicators and biomarkers. In this paper, we focus on the nexus between diet and MHSW in a population of Eco-Regions.

Eco-Regions conceptualise multi-actor governance with the overall aim to design a local sustainable food system that permits healthy and sustainable diets for all [3,7]. For citizens living in these regions, it may enhance their quality of life, also through positive changes in the natural and social environment, which eventually may influence both their somatic and mental health. Eco-Regions seem to function as best-practice examples of sustainable food systems as they provide favorable conditions for the development of different community areas (e.g., (agro-)tourism, organic agricultural practices, and community participation). INSUM has recognised a research gap in the area of food and nutrition security, quality and health of the citizens in the Eco-Regions. Current research on Eco-Regions has primarily focused on the organic approach and promotion of local value chains [4,8,9,10,11]. Therefore, INSUM aims to contribute to the development of knowledge about sociodemographic and health indicators in Eco-Regions. Health indicators must be distinguished from the term biomarkers. Dietary and health biomarkers are understood as indexes measuring the biochemical, cognitive or physiological changes in an exposed subject related to food or nutrient intake [12,13,14]. The term indicator takes a broader perspective that is not limited to the markers listed above.

This position paper elaborates on the main findings from the first INSUM workshop on MHSW. The three objectives of the workshop were (a) to present and to exchange on the current methodologies and indicators in the field of MHSW associated with diets, (b) to set these methodologies into the perspective of Eco-Regions and discuss potential study designs for populations of Eco-Regions and, (c) to create a network of experts with diverse backgrounds in the area of psychology, nutritional psychology and psychiatry, nutritional medicine, public health, child health and health science, toxicology, agriculture and health. Therefore, the guiding question for the workshop was:

*How can we test whether Eco-Regions’ communities and food systems which are more local, more sustainable and organic benefit MHSW of Eco-Regions’ citizens?*

Based on the scientific literature on MHSW indicators, experts with relevant knowledge to contribute to this debate were identified and ranked regarding their relevance within the project team. Workshop invitations were sent out to 56 experts. Twenty experts joined the workshop, comprising representatives of ten nations (i.e., Australia, Canada, Denmark, Germany, Netherlands, Norway, Poland, Sweden, United Kingdom (UK), United States (US)) and various disciplines (i.e., psychology of eating behavior, psychology of appetite, nutritional psychiatry, nutritional psychology, nutritional physiology, nutritional epidemiology, public health, health science, health psychology, agricultural methods and health, organic food systems). The 2-day hybrid workshop (online and on-site) was (organised and) hosted in Muenster, Germany, in May 2022. The workshop comprised presentations and discussion sessions. The discussions addressed the research design and the identification of potential indicators and tools to measure the MHSW effects of dietary transitions towards more sustainable and organic diets.

## 2. Research Background

### 2.1. Health

**Figure 1:**
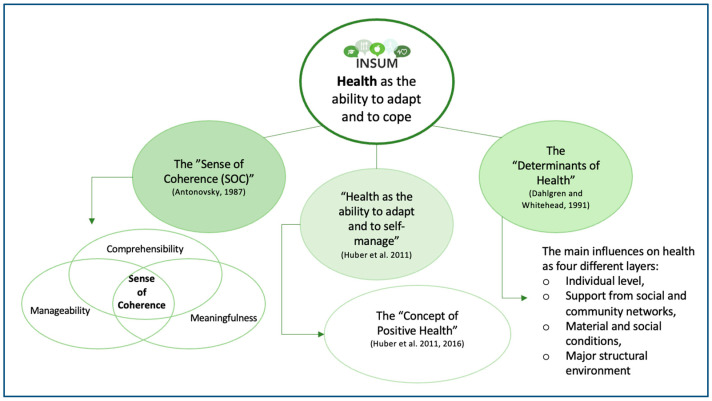
Current understanding of health within INSUM (in green) with the main influencing alternative health approaches [17,25,26]. The understanding of health within the project “Indicators for assessment of health effects of consumption of sustainable, organic school meals in Ecoregions” (INSUM) is built upon Aaron Antonovsky’s “Sense of Coherence” [26] (SOC), the “Determinants of Health” [25] and the “Concept of Positive Health” [19,20]. This current health approach may be further developed within INSUM’s progress (INSUM workshop 1 May 2022). Source: Authors’ own illustration.

The World Health Organization (WHO) [15] defines health as “a state of complete physical, mental and social wellbeing and not merely the absence of disease or infirmity”. The definition includes three domains of health. Next to somatic aspects, it emphasises the importance of mental and social wellbeing [16]. Upon publication, this definition was regarded as ground-breaking [16,17]. From our present point of view, it does not fit today’s society with its specific challenges as the WHO definition is static and considers health as a state (being healthy or not) [17]. The absoluteness of the word “complete” in relation to wellbeing neglects that one can feel healthy and live a meaningful life despite having, for instance, a chronic disease [16,18,19,20,21]. Then again, humans can be viewed as complex systems. From a systems perspective, health includes the ability to be able to deal with challenges and be resilient and adaptive. This is in line with the integrated character of Eco-Regions. For INSUM, the depicted health concepts (Figure 1) turn the focus from a state of wellbeing to health as a varying entity that can be individually adapted and influenced. The focus lies on the individual’s self-determination linked to external influences and the environment [20,21,22,23,24,25]. Thus, INSUM considers health as the individual’s ability to adapt and cope rather than health as an absence of diseases.

### 2.2. Mental Health

INSUM understands mental health and social wellbeing (MHSW) as dynamic states. There is a lack of consensus on a general definition of mental health. The following definitions presented are in line with the INSUM project’s aim: the WHO [27] defines mental health as the individuals’ awareness of their own capabilities and coping mechanisms that can be activated in case of external stressors. Furthermore, the ability to contribute to one’s community and work productively is emphasised. For the mental domain of their concept of positive health, Huber et al. [17] refer to Antonovsky’s “Sense of Coherence” theory which considers comprehensibility, manageability and meaningfulness as human assets that foster the individuals’ coping strategies and stress recovery. Accordingly, Keyes [28] builds on the salutogenic model and identifies three components of mental health: emotional, psychological and social wellbeing. Galderisi et al. [29] describe mental health as a dynamic state of internal balance, including a harmonious relationship of body and mind, the ability for social functioning as well as coping mechanisms to deal with inauspicious life events. In the field of positive psychology, shaped by Seligman [30], it is stated that five factors enable individuals and communities to flourish: positive emotions, engagement in life and work, relationships, meaning in life and work, and accomplishment. These definitions have commonalities, such as coping, wellbeing and social factors, including relationships or the community.

### 2.3. Social Health

The research on social or societal (referred to in the following as social) health and wellbeing is not a clearly demarcated field. Huber et al. [17] mention that several dimensions of health can be assigned to the social domain which highlights its broadness. Their social health approach is based on self-determination: the ability to fulfil one’s role in life—despite any medical condition—as well as being able to establish meaningful relations and experience a sense of coherence. Huber’s et al. [17] social health approach considers external conditions, like social and environmental challenges, which is of interest to Eco-Regions. Russell [31] also includes interactions and relationships with other people or social institutions. The WHO [32] puts forward the social determinants of health as non-medical factors, encompassing the environment and conditions where people are born, live or work that influence health and functioning.

Section 2.1, Section 2.2, Section 2.3 gave the workshop participants a broader view of health to challenge the traditional study designs on MHSW and diet.

### 2.4. Indicators and Assessment Methods

Several indicators and methods of MHSW have been used in dietary studies. We differentiate indicators from methods in the following way: an indicator refers to an index measuring certain outcomes and methods comprise the procedure to assess the indicator, including practical tools applied in current research. Dietary studies predominantly rely on disease risk markers. MHSW and diet is still an emerging field, esp. with a focus on health that supports INSUMs understanding of health (see Section 2.1, Section 2.2, Section 2.3).

Three reviews provide an overview of the diverse indicators and methods previously used [33,34,35]. Van Dyke and Drinkwater [33] carried out a literature review on the relationship between intuitive eating and health indicators and grouped the indicators into two domains, physical and psychological. They identified, for instance, self-esteem, satisfaction with life, or optimism as psychological health indicators. Furthermore, Głąbska et al. [34] conducted a systematic review of fruit and vegetable intake and its relation to mental health in adults and presented several assessments (indicators) and psychological measures from the reviewed studies. Focussing on a younger population group, O’Neil et al. [35] conducted a systematic review of the relationship between diet and mental health in children and adolescents. They identified various instruments to study mental health and assess dietary patterns, such as the Emotional Symptoms Subscale of Strength and Difficulties Questionnaire (SDQ) (Parent) or Health records of physician-diagnosed internalizing disorders (ICD-9) (child), both combined with Food Frequency Questionnaires (FFQ). A further review targeting youth was performed by Dimov et al. [36]. They used the SDQ in combination with a six-item screening tool to assess the associations between diet quality and mental health problems in late childhood. Moreover, Puloka et al. [37] analysed dietary behaviours and the mental wellbeing of New Zealand adolescents. On the basis of data from a national survey on health and wellbeing, they applied the WHO Well-Being index, the Reynold’s Adolescents Depression Scale and the SDQ together with Eating Behaviour Questionnaires. The two domains, MHSW, were brought into relation with different lifestyle factors by van Lente et al. [38]. They applied the 14-point Oslo Social Support Scale and utilised data from a national survey of lifestyles, attitudes and nutrition to determine population MHSW in relation to different lifestyle factors. Social parameters associated with diet quality were analysed by Hoerster et al. [39]. The study focussed on social support and neighbourhood factors and combined the 8-item Starting the Conversation tool with, amongst others, the ENRICHD Social Support Instrument to measure diet quality and social support.

### 2.5. Sustainable Healthy Diets and Organic Principles

Health associated with food consumption is defined as an integrated part of food systems. ‘Sustainable Healthy Diets’ encompass three components: (1) health and wellbeing; (2) low environmental pressure and impact; (3) food that is accessible, affordable, safe, and equitable, as well as culturally agreeable [40]. Organics International describes health in one of their four leading principles as “... the wholeness and integrity of the living systems” with characteristics of resilience, immunity and regeneration [41]. The latter concept of recognising health as an integrated part of the health of ecosystems is also reflected in the One Health approach. One Health links the health of people, animals and ecosystems [42].

Consumers state health as one of their major motivations to choose organic food, such as the environment, animal welfare and taste [43]. The organic food market is expanding and consumers’ demand for organic food is increasing worldwide [44]. In the European Union (EU), organic food production is regulated by the Council Regulation (EC) 2018/848 [45]. According to the EU’s Action Plan [45], organic farming is crucial for the production of high-quality food with low environmental impact [45]. The EU legislative [45] specifies among others that organic crops are cultivated without the use of synthetic chemical plant protection agents (insecticides, herbicides and fungicides), highly soluble synthetic nitrogen, phosphorus and potassium fertilizers, and synthetic growth regulators.

Eco-Regions meet the international political sustainability agenda. On the EU level, Eco-Regions cohere to the EU Green Deal (i.e., 25% organic land in the EU by 2030), EU Organic Action Plan (i.e., Action 14) and EU long-term Vision for Rural Areas [46,47,48]. Internationally, Eco-Regions are embedded in several United Nation’s (UN) Sustainable Development Goals (SDGs), i.e., SDG 2 (zero hunger), SDG 3 (good health and wellbeing) and SDG 11 (sustainable cities and communities) [5,49].

Several scholars indicate that organic food may positively influence health outcomes (e.g., cancers, metabolic syndrome) [50,51]. Organic diets were correlated with various indicators of a healthy lifestyle, and, among others, with a lower obesity incidence [52,53]. A cross-sectional study from the French Nutrinet-Santé cohort indicates that individuals who had a higher intake of organic products also had a healthier diet according to Food-Based Dietary Guidelines, such as organic food consumers were likely to eat more plant-based products [54,55]. The aforementioned studies point to positive health effects related to organic diets. However, it remains unclear whether these are a direct effect of the organic diet, the reduced levels of contaminants or if more health-conscious people prefer organic diets in the first place. Thus, further research needs to be done on potential positive health effects and their underlying factors [56].

Many studies that address organic diets have focus on classical disease-risk biomarkers in common [52,53,57,58]. However, the effects of dietary changes towards more sustainable diets can at the same time influence psycho-social aspects as well as the metabolome or microbiome. In the 8-week Randomised Controlled Trial (RCT) study, the effect of a Mediterranean diet on metabolic health is assessed in a group of overweight and obese subjects. Multiple changes were seen in the microbiome and the metabolome while the only classical health marker affected was blood cholesterol [59]. To the authors’ knowledge, hitherto MHSW has not been examined in connection with organic or sustainable diets.

## 3. MHSW Research Related to Diets

The workshop discussions were based on the following research domains: public health, nutritional psychiatry, nutritional psychology, nutritional epidemiology, psychology of eating and influences of social contexts in food choices and intake. The main points presented at the Workshop are summarised, which include the approach of Positive Health and measurements targeting diets and health in multiple population groups.

### 3.1. Positive Health

Dr. Marja van Vliet opened the presentations by introducing the concept of Positive Health. As a response to the static WHO definition of health [60], Huber et al. proposed a new dynamic concept of health: ‘health as the ability to adapt and self manage’ [17]. They were inspired by an earlier research project in which chicken fed with organic food showed to be better able to overcome immunological challenges compared to chicken fed by a conventional diet [61]. Based on the WHO definition, it was impossible to conclude whether this response reflected a healthier condition.

For public health purposes, the new, dynamic concept of health was operationalized into the concept of Positive Health. Positive Health encompasses six dimensions (bodily functions, mental wellbeing, meaningfulness, quality of life, social and societal participation and daily functioning) [62]. Overall, it aims to stimulate resilience and contribute to achieving a meaningful life. As a practical method, the Positive Health dialogue tool was developed [20,63].

Positive Health has become a catalyst in the Dutch public health domain by stimulating health promotional initiatives that focus on a broad range of health aspects and in which people take centre stage [20] Subsequently, appropriate indicators are required to determine the beneficial effects. To fill this gap, research is conducted to transform the Positive Health dialogue tool into a Positive Health measurement scale [63,64]. Given the overlap between Positive Health and the rationale of sustainable healthy diets and organic principles, Positive Health seems to serve well as a framework for the INSUM project. Furthermore, the Positive Health measurement scale which is in development can provide clues for suitable indicators to measure mental and social health.

### 3.2. Nutritional Psychiatry and Psychology

Prof. Adrienne O’Neil and Dr. Wolfgang Marx contributed to knowledge about the research area of Nutritional Psychiatry. This emerging field has provided multiple lines of evidence that indicates that diet quality is an important lifestyle factor in the management and prevention of depression [65]. Meta-analyses of several prospective and cross-sectional observational studies have reported that improved diet quality is associated with a reduced risk of depression [66,67]. This association appears to be consistent across multiple datasets, throughout the lifespan, and is apparent using multiple diet quality indices [66,67,68]. Furthermore, mechanistic studies have identified multiple pathways whereby diet may modulate depression pathophysiology with much of the recent research focusing on the role of diet in the modulation of the gut microbiota-brain axis [69,70]. Importantly, a meta-analysis of randomized controlled trials suggests that dietary interventions can improve depressive symptoms and there is now a growing number of randomized controlled trials that report similar improvements in people with clinical depression [71,72,73]. Due to the growing evidence base to support the use of dietary interventions, this is now being reflected in various guidelines including the 2020 Royal Australian and New Zealand College of Psychiatry guidelines [74].

Ephimia Morphew-Lu defined the diet-mental health relationship (DMHR) as a concept of how humans think, behave, feel and experience in the context of nutrition. Nutritional psychology is the growing field of a study aiming to characterise the DMHR: more specifically, the study of behaviour [75], cognition [76], sensation [77], perception [78], psychosocial factors [79], interoception [80] and psychological health, mood, and well-being [81,82] in relation to diet. A growing body of empirical evidence illustrates the ways in which dietary intake affects psychological functioning and mental health [70,81,82,83,84].

Some indicators used in DMHR research include executive functioning (e.g., neurocognitive testing) [85,86], well-being and pleasure (e.g., Warwick-Edinburgh Mental Well-being Scale; Control, Autonomy, Satisfaction, Pleasure Scale; Anticipatory and Consummatory Eating Pleasure Scale) [87,88,89], behaviour (e.g., Three-Factor Eating Questionnaire) [90], resilience (e.g., Simplified Resilience Score) [91], sleep (e.g., Pittsburgh Sleep Quality Index) [92], and environment (e.g., Nature Relatedness Scale) [93].

### 3.3. Indicators and Tools for (Mental) Health Outcomes

Prof Ailsa Welch gave an overview of different nutritional indicators, or biomarkers, for the assessment of general health effects, besides MHSW. Dietary surveys to determine nutritional intake in population groups often use classic assessment methods such as 24 h recalls. However, nutritional biomarkers are potential alternatives for measuring nutritional status. Tissue sources of biomarkers range from saliva to blood or urine samples. Although nutritional biomarkers have advantage of being independent of the measurement error that occurs with dietary reporting, homoeostatic, physiological and lifestyle factors influence the accuracy of their measurement, e.g., smoking habits, sex and body mass index (BMI) interact with circulating concentrations of vitamin C [94].

There are limited biomarkers for macronutrient intake. However, quantitative recovery biomarkers collected over 24 h such as urinary nitrogen excretion, a measure of protein intake, are well established. Concentration markers measured in the blood can only reflect some of the nutrients as others are highly regulated by homeostasis. However, a number of markers exist including the vitamins C, E, D, B_12_, carotenoids, iron and circulating n-3 polyunsaturated fatty acids, which reflect the intake of fish [95].

Newer developments in nutritional biomarkers include the measurement of magnesium status in skeletal muscle using magnetic resonance imaging [96], urinary metabolites/metabolomics which validates consumption of specific food groups [97], and measurements of carotenoids in the skin using spectroscopy. Predictive biomarkers such as urinary measures of sodium, fructose, sucrose, and pH also hold promise [98,99]. However, sample collection for many biomarkers is intrusive, leading to issues with their use, particularly in children.

Dr. Richard Peter Hayhoe pinpointed the increasing prevalence of poor mental wellbeing in children and young people that is of concern [100]. Together with a group of scientists, Hayhoe sought to determine the association of nutrition, specifically breakfast and lunch meal choices, and fruit and vegetable consumption, with mental wellbeing in schoolchildren in Norfolk, UK [101]. Data from over 10,000 pupils were collected via questionnaires at 56 schools and colleges. Mental wellbeing was assessed using validated measures: the Stirling Children’s Wellbeing Scale [102] for younger (primary-school) children and the Warwick-Edinburgh Mental Wellbeing Scale [88] for older (secondary-school) children. Nutrition and relevant behaviour, health, and demographic data were also collected. Multivariable linear regression was used to test the association between nutrition variables and mental wellbeing while adjusting for covariates. In secondary-school analyses, higher fruit and vegetable consumption were associated with higher wellbeing scores while nutritionally poor meal choices at breakfast or lunch were associated with lower wellbeing. In primary-school analyses, similar associations were seen between meal choices and mental wellbeing [101]. These findings support the assumption that good nutrition is important for the mental wellbeing of children and young people.

### 3.4. Food Choices and Social Context

Prof Suzanne Higgs gave insights into the research on the influences of social contexts on food choice and food intake (i.e., food environment) [103]. Social context exerts a pervasive and powerful influence on what and how much we eat [104]. Social factors also affect how we connect with others around food and influence wellbeing. People who eat together more frequently have larger support networks, feel more connected with their community and are more satisfied with life [103]. Sharing food also promotes trust and cooperation [105]. Food choices are furthermore related to our social identity and how we relate to other people [106]. Identifying oneself as a person who cares about the environment is related to an increased purchase of organic food and a greater feeling of connectedness with other organic food consumers [107]. Taken together these data suggest that communities that are working together to transition towards more sustainable food systems may experience benefits in terms of improved wellbeing and more cohesive social relations.

## 4. Workshop Results and Discussion

The discussion comprised socioeconomic factors and potential research designs, encompassing the object of study as regards the region and the time period, and the population group as well as appropriate (combinations of) measurements.

### 4.1. Socioeconomic Factors and the Food Environment

Discussions identified important socioeconomic factors related to the available food environment, including access, knowledge and affordability of healthy and organic foods. In the example of the UK, it was highlighted that organic food is primarily purchased by higher social classes with higher education [108]. At the same time, it was mentioned that, currently, food banks are experiencing a higher demand [109,110]. Therefore, access to (healthy) food, in general, was a concern that was raised but also a lack of knowledge of healthy and organic foods. Consequently, arguments were given for the reasons for the current higher costs of organic food and how these costs could be reduced and at the same time address the access to healthier foods. In this sense, the internalisation of external costs was mentioned. The current prices of foods do not indicate the “true costs”, as environmental costs for production are not included in the prices [111]. Food waste needs to be tackled as well as a shift from the production and consumption of meat to more vegetables. In addition, successful practices from other countries were expressed. One expert mentioned that informal discussions with local stakeholders in Eco-Regions indicated that organic food, with the example of the bio-district Cilento, Italy, is largely accessible to the local population. However, research is needed to verify this statement. Sweden aims at becoming 60 percent organically in public procurement by 2030 [112]. In Copenhagen, the political goal for public procurement is to reach an organic share of 90 percent and has so far reached 87 percent [113,114]. In this sense, the importance of leading roles, support and education was stressed and potentially also the mindset and consciousness of consumers.

Understanding dietary recommendations and the food environment was a matter of interest. Following guidelines on healthy diets is not straightforward for a large proportion of the population [115,116], e.g., the “5-a-day” in the UK to increase the intake of vegetables and fruits as, for instance, the understanding of serving sizes differ, especially for the youngest [117,118]. In addition, social norms, eating stereotypes and social identity are influencing dietary intake (cf. vegetarianism rated as less masculine than an omnivore diet). Shifting to more sustainable diets, including less meat and less animal-based foods might be seen as a threat to the individual identity [118]. Thereby the question arose of how these findings can be translated into interventions to protect people from their perceptions and cultural identity (cf. high meat consumption as masculine) [118]. In this context, food as a connecting factor to other lifestyle dimensions was emphasised and each individual’s food choices are a reflection of the surrounding environment, like culture, home or income [106,107], among other determining factors such as education or sex. Research on the Social Determinants of Health underscores the necessity to consider education, income, ethnicity, social status, housing, etc. as mediating factors for health [119]. Furthermore, one expert mentioned that the concept of connectedness, to other people, the neighbourhood and to themselves, gives meaning to life and eventually influences health [120] which is why focusing on Eco-Regions offers the opportunity to include those surrounding factors within a specified area.

The experts concluded that the role of public meals, especially healthy school meals, is a possibility to reach people across social classes, especially the youngest. Participation in the preparation of school meals can increase awareness and knowledge of organic and healthy foods [121,122], including less animal and more plant-based food and the prevention of food waste. Thereby, a first potential setting was identified, where future research on influences of more sustainable and organic diets could take place.

### 4.2. Research Design

Procedures and methods are specified in the research design. Any study design can vary in its structure and is dependent on the research aim [123,124]. During the workshop, different aspects were deciphered that fall under the scope of the research design. The discussion comprised the study object including a future-study time period, regional aspects, types of studies and assessment approaches as well as potential population groups.

#### 4.2.1. Region and Time Period

One topic of interest was the region where future research could take place. A few workshop participants suggested a comparison of different regions, namely Eco-Regions being in different stages, e.g., established versus new evolving Eco-Regions or regions where a Mediterranean diet or traditional indigenous diets are followed. One expert shed light on geographic differences that need to be taken into account, e.g., urban and rural settings, the climate and its impact on mood and health when comparing southern and northern countries. Socioeconomic differences between nations also need to be considered.

Among the workshop participants, there was a general agreement for the need for longitudinal studies, proposing cohort studies as one potential approach. One reason for this idea was to enable the detection of small differences in human MHSW that an organic diet can potentially have over several years. The length of the period was left open for further discussion. Different regions with distinct development stages could be monitored over a period of time and MHSW differences measured between communities in established and evolving Eco-Regions.

The discussion went further on to measuring-possibilities of sustainability in Eco-Regions. A future research design may consider the conceivable advantages of an Eco-Region, for instance, in the environmental domain, compared to non-eco-environments (e.g., via Life Cycle Assessment). For Eco-Regions in different development stages, potential change effects, such as in agricultural practices and community lifestyles, could be of interest to be observed. Accordingly, the current stage and baseline of each Eco-Region that will be selected as a case study need to be assessed [120,121]. In this sense, indexes on sustainable and healthy dietary intake, similar to the healthy eating index [125], were proposed. This also includes whether the food products included in a diet are produced in a more sustainable way.

#### 4.2.2. Study Population (Adolescents, Children, Adults, Families)

The experts discussed distinct constellations of study objects. Any age group could be selected as a study object and it seems beneficial to go beyond schoolchildren and the previously mentioned school meals and include multiple groups (i.e., schools, families). Healthy and sustainable school meals together with food and nutrition education may provide a spill-over effect to the family or community [126]. For INSUM it seems beneficial to re-address the narrow way we look at the population of Eco-Regions. The concept of One Health addresses a human’s interconnections with all that surrounds us, especially in regard to the environment as well as human and animal health [42]. This fact could be relevant to consider for the assessment of MHSW and diets in Eco-Regions where the environment and nature (organic agriculture) are the subjects of the community [127].

Experts reported that the interest in children especially rises from the need to understand and monitor children’s development processes. Several studies have been conducted on children’s eating behaviours [128].

The experience by experts highlighted that school settings typically include one of the target groups: children of young age (primary school), adolescents (secondary school), and/or parents [101].

Experts argued to include **young children** under the age of 14 years, an age where many behavioural conditions are formed [129]. Further, potential exposure to environmental toxins (e.g., PFAS), as somatic health indicators, can be assessed from an early age [130]. Workshop experts discussed some ongoing shifts in young-aged children [131,132,133] with a tendency of more “adolescent-like” behaviour at an earlier stage of life: “As for so many health conditions, we begin to realise that things begin to develop much earlier” [citation from expert, 2022]. In terms of mental health, presumably, children at a young age do not know how to overcome group pressure. Therefore, it was proposed to conduct a cohort study with young age groups to assess changes over time.

In addition, many experts argued that **adolescence** is an interesting period of life for the assessment of MHSW [35,134,135,136,137]. Adolescents gain a certain amount of independence, including food choices [138,139]. Adolescents experience a change of identity. In this stage of life, peer influences are strong and family relations play a role [140,141,142,143]. It was highlighted that the influences of these changes on MHSW represent an interesting field of research. Therefore, the experts proposed to include a range of adolescents (e.g., between 10–18 years) with the advantage to assess the changes in adolescents’ social identity related to food and eating behaviour and perceptions over time. Another advantage is that questionnaires for adolescents do not require an adaptation—questionnaires for adults could be applied directly.

**Parents** could be a relevant study population to assess both the MHSW of their children and themselves. Research shows the parental influence on children’s eating habits and vice versa [144]. Parental awareness is crucial for the dietary development of their children. For instance, an influence was shown in the long-term intake of poor diets that may contribute to a higher risk of diseases [115]. Parents also facilitate children’s eating situations in which stress and anxiety can impact both child and parental psychological wellbeing [145]. Therefore, the combination of both children and parents as study objects, is of large interest for INSUM, to interconnect the private surroundings and school meal settings. Assessing **families** has the potential to combine several perspectives [131]. How children perceive their own health is also connected to family interactions. For surveys, it is beneficial to ask both adults and children (different perspectives). This setting would also allow gaining further background knowledge, for instance, about the family’s lifestyle, including the purchase of food, (i.e., retail vs. farmers market), food preparation, sleeping patterns, motives to support their children with school meals or not and other societal aspects. School settings allow for setting the boundaries of the study object. Another benefit of including children is also that this study may contribute to doing something for the children that can affect their future health over their lifetime.

### 4.3. Measurements and Indicators for the Study on MHSW

**Figure 2:**
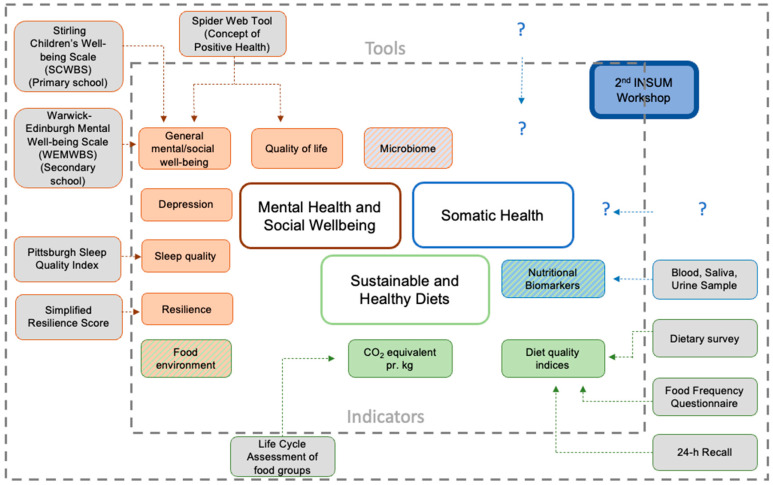
Results of discussed indicators and tools to assess mental health and social wellbeing on diet (INSUM workshop 1, 2022). The inner square boundary shows indicators for the three categories: Mental Health and Social Wellbeing (red), Sustainable and Healthy Diets (green), Somatic Health (blue). The mixed-colour boxes symbolise indicators that can be related to more than one category. The outer part displays tools for the assessment of those indicators. Not all indicators were discussed in relation to specific tools. This figure raises no claim for completeness. It serves rather as on overview of discussed indicators and tools. This figure reflects the main results of the first INSUM workshop and can be understood as a continuous developing toolbox (e.g., INSUM workshop 2 on somatic health works on the somatic health area (i.e. “?”)). The results of the discussion showed that a “battery of tools” (i.e., combination of different tools from distinct domains) is needed to assess the complex system of human health.

As a result of the presentations and discussions, Figure 2 represents an overview of indicators and tools for future research on the effects of dietary changes on MHSW. During the workshop, somatic health indicators were a matter of interest, emphasising the interrelatedness of the different health domains. Due to the complexity of each domain, somatic health indicators will be the topic of interest in the second stage of the project INSUM. Therefore, the figure will be further developed within INSUM’s progress.

The discussion unveiled concerns about how to measure the effect of diet in healthy study participants. This led to proposals of combining different research methods (mixed methods research [146]), such as interventional next to observational studies. Qualitative and quantitative measurements could be combined with investigations in living labs (i.e., Eco-Regions). Experimental studies within longer-term trials were suggested to see the short- and long-term effects of organic diets. Randomised controlled trials (RCTs) [147] were a matter of interest when it comes to determining causalities between organic diets and health. However, it was concluded that firstly research should aim at identifying the particular parts where changes in health can be expected before RCTs can be introduced to verify these indicators. In this aspect, one participant referred to the French NutriNet-Santé cohort study [54]. The study uses statistical tools to adjust for the impact of lifestyle factors in the comparison of organic vs. non-organic diets and their influences on health.

Experts mentioned microbiome research as one of the areas of potential focus combining mental and somatic health [83,148,149], as current research shows an increasing incidence of the bidirectional communication between the central nervous system and gut microbiota (the gut-brain-axis) [150]. The level of resilience was discussed as a potential indicator for MHSW that could be measured by reactions or coping strategies to certain kinds of challenges in a sub-group over time. It was highlighted by the participants that MHSW and somatic health are interacting closely in the complex system of human health and therefore cannot be assessed separately. For INSUM, in addition to qualitative mental health questionnaires, the participants pointed out that somatic health indicators can be useful to prove the health status quantitatively. Therefore, the two domains should be seen as complementary and both included in research on the health effects of more sustainable and organic diets.

### 4.4. Questionnaires to Assess MHSW

The workshop participants had a general consensus on including questionnaires to monitor MHSW outcomes. Several strengths and weaknesses of questionnaires to assess MHSW related to diets were discussed.

On the one hand, three potential strengths were identified: first, the possibility to combine several aspects in one tool [151], such as wellbeing, coping mechanisms and lifestyle. Second, validated questionnaires for adults can largely be applied to adolescents [152]. Third, questionnaires have the possibility to include diverse interest groups, such as parents, teachers, and children [153]. On the other hand, three potential weaknesses came to the fore: first, comparability between different cultural settings [154], second, translations [155,156] and their influence on the meaning of MHSW questions, and third, the distribution [157]. For an international study consortium, comparability is key but can be challenging for diets, because cultural food and eating habits play a large role. Several diet-disease research questionnaires, such as the food choice questionnaire (FCQ) and food frequency questionnaires (FFQ), are validated across borders [154,158]. However, the validated application across national borders is not known for any of the MHSW questionnaires presented in the INSUM workshop.

Experts agreed that especially for MHSW investigations, the scope of questions is crucial to maintain the meaning. Therefore, translations of questionnaires can pose a risk and would require at least an appropriate validation [155,156].

The distribution of questionnaires is also important. Social influences and peer pressure can also impact the quality of results [157]. For instance, pupils who answer the questions in a group setting instead of individually. Peer pressure could be a potential factor to consider, especially when the distribution of questionnaires is left open to the schools as it was done in the UK Study of Norfolk teenage children [101]. Therefore, the distribution of the questionnaire is impacting the outcome and hence, needs to be considered beforehand.

### 4.5. Workshop Strengths and Limitations

Overall, a strength of the workshop is the open and explorative approach as the research on MHSW in relation to diet is still developing and there is not much known yet in association with more sustainable and organic diets. Further, the discussion group consisted of diverse experts. Such an interdisciplinary and international gathering can contribute to finding new ways to conduct research on sustainable development. The invited experts covered expertise ranging from sustainable food systems, over somatic health, and agriculture to MHSW. In addition, the hybrid format enabled experts from different countries (e.g., the US, Canada, Australia) to participate. The narrowed number of experts can be seen as a limitation of the workshop that potentially did not allow the gathering of all important information. At the same time, the limited number supported a direct discussion within the whole group where everyone was heard. Further, among the participants, high-income countries were overrepresented. This could be seen as a limitation since the global food system is not covered. Many low-income countries are especially challenged by environmental stresses (e.g., droughts, lack of drinkable water) and access to healthy food.

Taking all the pros and cons together, the workshop set the base for interdisciplinary collaborations and the development of explorative research designs. These are demanded to meet the needs of regions affiliated with sustainable development (e.g., Eco-Regions), including the effects of diet on MHSW.

## 5. Conclusions

The first INSUM workshop discussed how it can be tested whether Eco-Regions’ communities and food systems benefit the MHSW of Eco-Regions’ citizens. Eco-Regions seem like a manifold community concept for a more sustainable way of living. The participants concluded that a “battery of indicators” will be needed to assess the health effects of living in regions devoted to sustainable development (cf. Eco-Regions). One set of indicators can be utilised for everyday monitoring and a further set for in-depth detailed research. A combination with quantitative somatic health indicators and tools has the potential for complementing the more qualitative MHSW measurements and indicators.

The workshop concluded that MHSW and somatic health cannot be assessed as two separate entities. Thus, human health is a complex interacting system. As per definition, being mentally and socially well relies on the presence of resources, including coping mechanisms to deal with adverse life events, the ability to adapt as well as the possibility to fulfil one’s life in a meaningful way. This also entails engagement and interaction with the community and other people.

The interdisciplinary group of experts discussed three relevant topics: socioeconomic factors, research designs and tools as well as their strengths and limitations. The complexity of the research domain and the explorative approach led to a shift from the focus on concrete MHSW indicators towards a debate on the research design on a broader level.

The discussion on socioeconomic factors including access to and affordability and knowledge of healthy and organic foods led to the conclusion that a food systems approach is needed where sustainable and organic foods along with healthy meals are part of the system, e.g., in public procurement. To which extent these factors should be included in future research in Eco-Regions was left open as it at least can be assumed that access to organic food is ensured in those regions. Eating stereotypes and social identity are influencing dietary intake and therefore were identified as factors that need to be included when conducting research on MHSW.

A consensus was reached to collect data in three different Eco-Regions of varying stages (newly evolving, established) as living labs with a cohort over a longer period in combination with interventional and/or experimental studies. For integrated projects such as INSUM, it is a challenge to reveal the process of change, for instance, the food transformation process and improvements in health over time, which is why living labs seem appropriate. From the discussions, it arose that school meals not only affect children but may also influence their surroundings, including family members. Therefore, the research object family seems adequate to address this complex research in Eco-Regions on MHSW as diverse interrelations (parents, school, and children) can be monitored.

The choice of tools remains rather limited to questionnaires when investigating the MHSW of a cohort, but the effect of those can be quite different depending on the research design or distribution channels (e.g., group setting and peer pressure). When assessing mental health with questionnaires, the formulation of questions is essential for the outcome. This sensitivity must be considered when designing an international cohort study. Translations may limit the validity of results but also cultural and regional differences. The utilisation of nationally validated questionnaires and tools is regarded as a reasonable option if the beforehand mentioned limitations are considered.

The research on health outcomes of regions devoted to sustainable development, including sustainable, healthy and organic diets, is still a developing field. During the workshop, several factors were addressed that are influencing the research and at the same time, new questions evolved. Five aspects were striking and need to be taken into account in future discussions. First, the measurement of changes in already healthy participants that are shifting to a more sustainable and organic diet. Second, the identification of indicators to assess even the very small differences between these shifts in health. Then, the adjustment of confounders and lifestyle factors in regional settings when research focuses primarily on the dietary impacts. Fourth, it must be ensured that questionnaires are validated and can be used in an international research context covering cultural and regional differences. Lastly, how the somatic domain can be further integrated into the research and which tools and indicators might be useful. To explore health as a whole, a second workshop will be facilitated on the somatic health effects of dietary transitions towards more sustainable and organic diets. The outcomes of both workshops can set the baseline to initiate interdisciplinary research about the potential health effects of living in Eco-Regions.

## Data Availability

The original contributions presented in the study are included in the article.
